# Supplementary material for: Bacterial hopping and trapping in porous media
Source: Nat Commun. 2019 May 6;10:2075. doi: 10.1038/s41467-019-10115-1 (PMC6502825; doi:10.1038/s41467-019-10115-1)
Supplement: Supplementary file 1 — Supplementary Information [file 41467_2019_10115_MOESM1_ESM.pdf]

Supplementary Information:
Bacterial hopping and trapping in porous media

T. Bhattacharjee and S. S. Datta

This file contains Supplementary Figures 1-11.

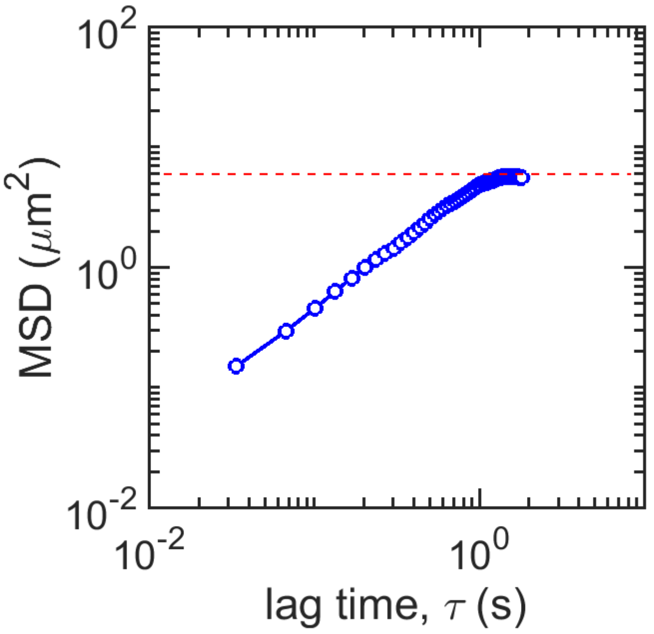

Supplementary Figure 1. Pore size measurement using MSD of dispersed nanoparticles. Figure shows representative MSD of a tracer particle. Measuring the length scale at which the tracer MSD plateaus ie. becomes independent of the lag time provides a measure of the smallest confining pore size of the medium.

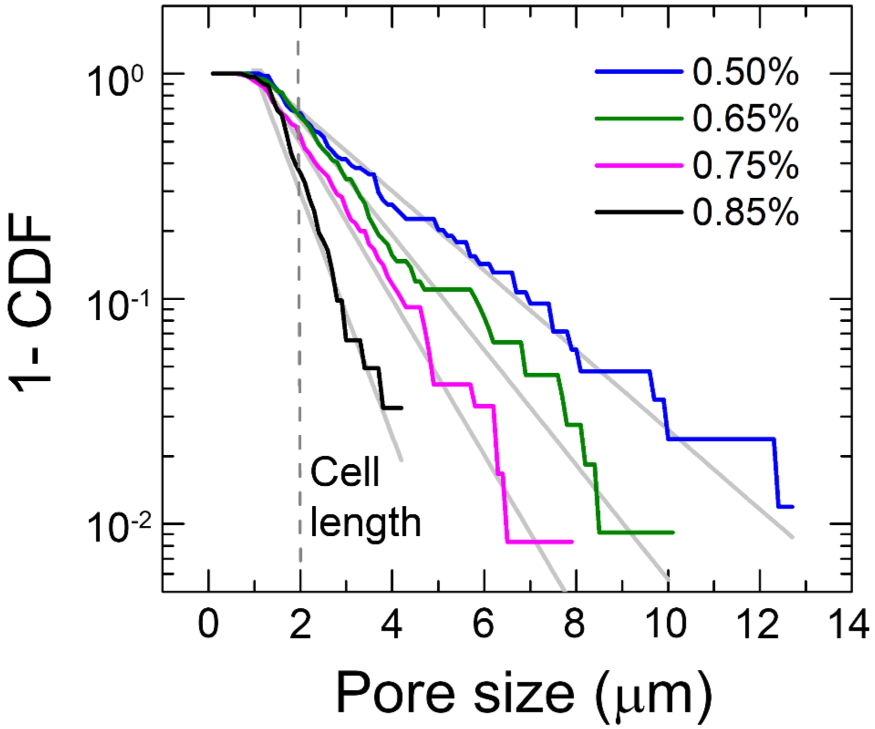

Supplementary Figure 2. Exponential pore size distribution. We fit an exponential function B*exp(-a/A) to the 1-CDF data in the range of 0 < CDF <1. We report the characteristic pore size as ($A+a_0$), where a_0 is the largest pore size with CDF = 0.

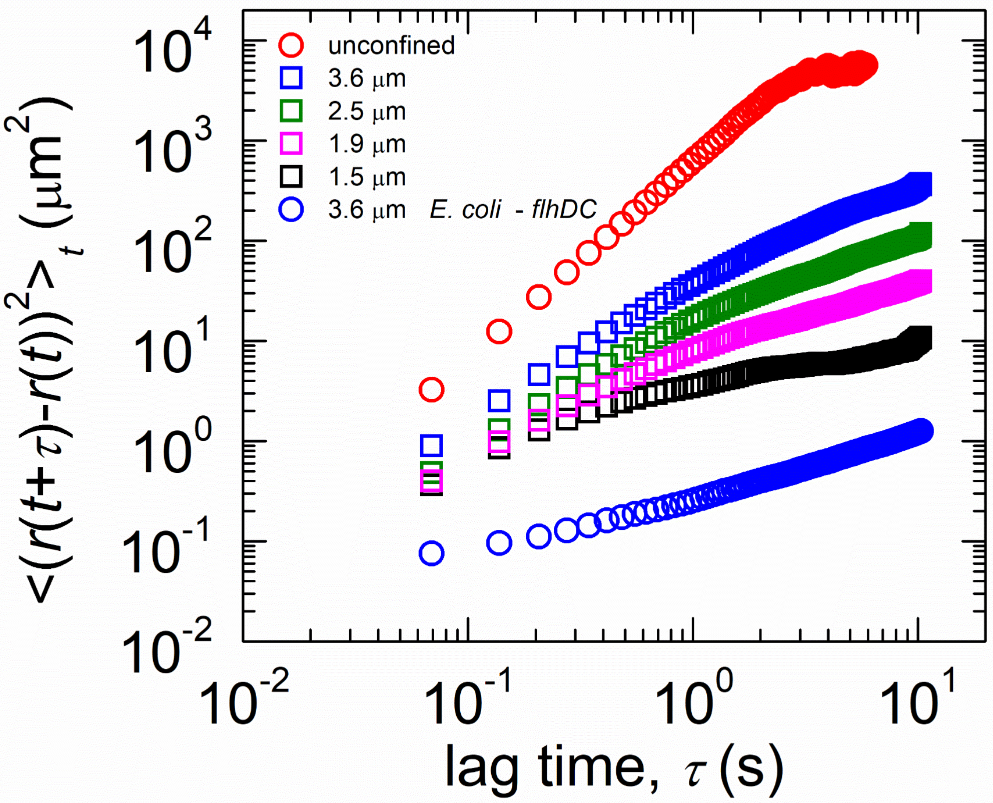

Supplementary Figure 3. Motility of mutant *E. coli* inside porous media. We track the motion of a mutant *E. coli* strain that is not capable of assembling flagella (blue circles) in porous media with a characteristic pore size of 3.6 μm. From the ensemble average mean squared displacement, we find that their motion is negligible compared to their wild type counterparts (squares); the MSD of the mutant is an order of magnitude smaller at the shortest lag times, and over two orders of magnitude smaller at the largest lag times, than the wild type in the same porous media (blue squares).

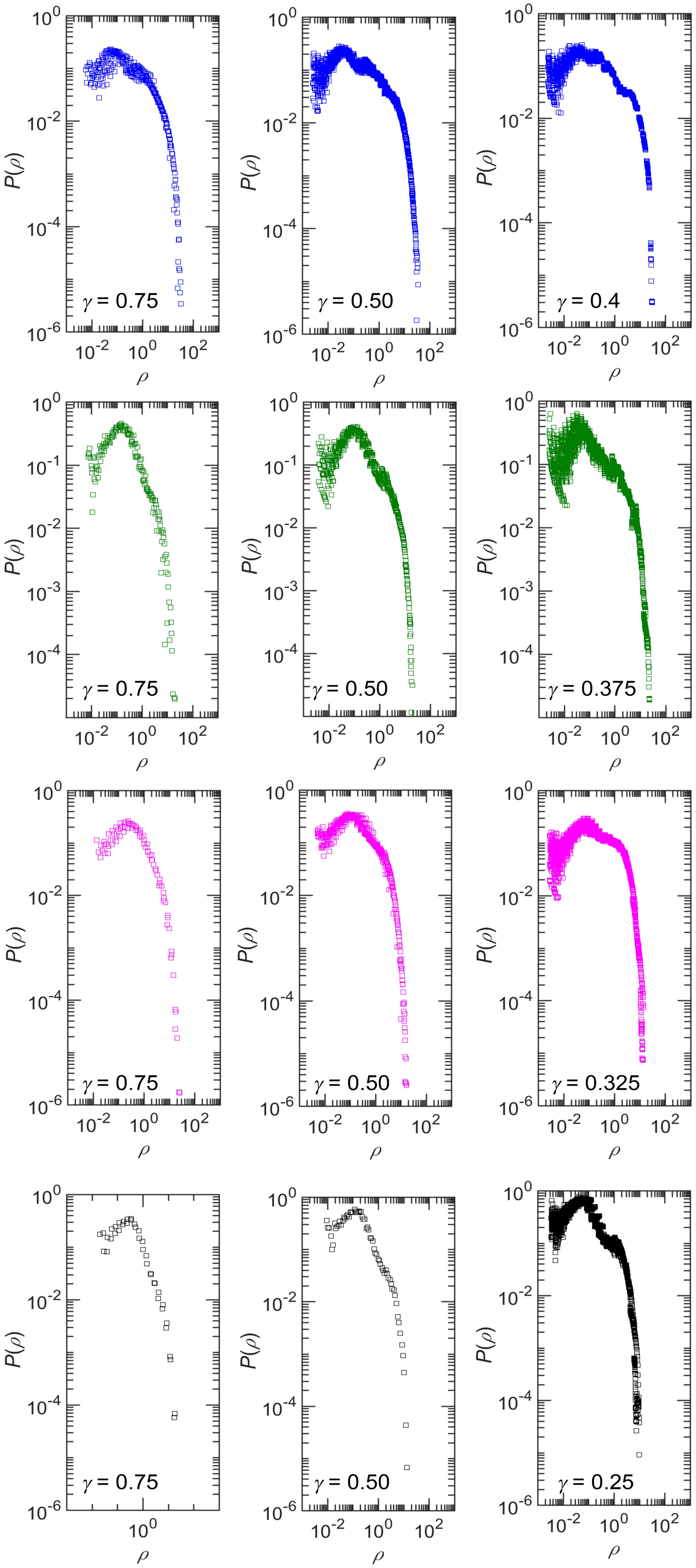

Supplementary Figure 4. Step size distributions. To classify the motion of bacteria cells in porous media, we investigate the distance travelled by each cell as a function of increasing lag time, τ. At every lag time, a step size distribution, $P(r)$, is computed as piecewise constant probability density functions for all cells throughout all experimental times. To further verify if there is any transition in bacteria motility, we plot the normalized step size distribution, $P(\rho)$, where ρ is defined as $\rho \equiv r/\tau^{\gamma}$; the exponent γ indicates different classes of motion. $\gamma = 0.5$ captures diffusive motion whereas $\gamma > 0.5$ indicates super-diffusive behavior and $\gamma < 0.5$ represents sub-diffusive motion. For bacteria migrating in different porous media (colors represent different porous media as given in the legend of Supplementary Fig. 3), we find that the normalized step size distributions collapse to single curves at different regimes of τ. Three regimes of motion—super-diffusive, diffusive and sub-diffusive—are observed with $\gamma = 0.75$ for all super-diffusive regimes. For the sub-diffusive regimes of motion, γ decreases with the decrease in pore size indicating prolonged trapping of bacteria in confinements.

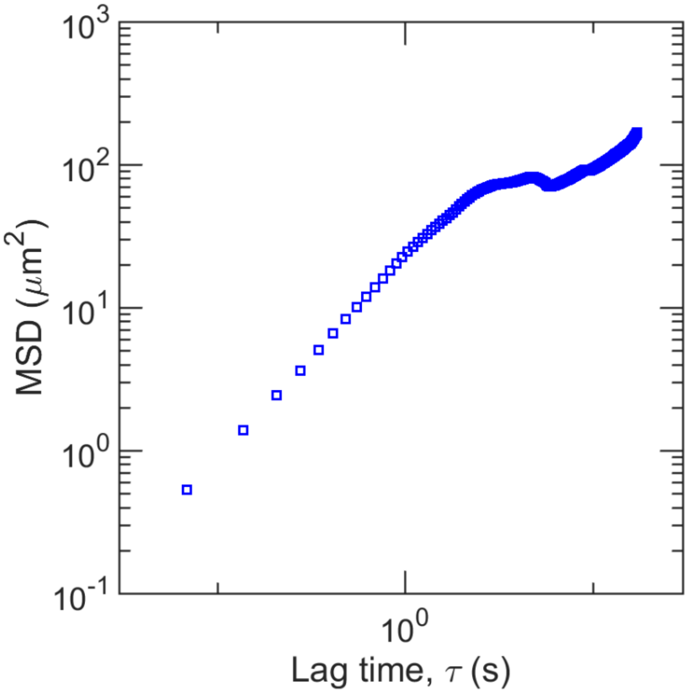

Supplementary Figure 5. Single cell MSD. Mean squared displacement of a single cell inside a porous medium with a characteristic pore size of 3.6 μm, showing that at long timescales the motion of individual cells becomes diffusive after transient subdiffusion.

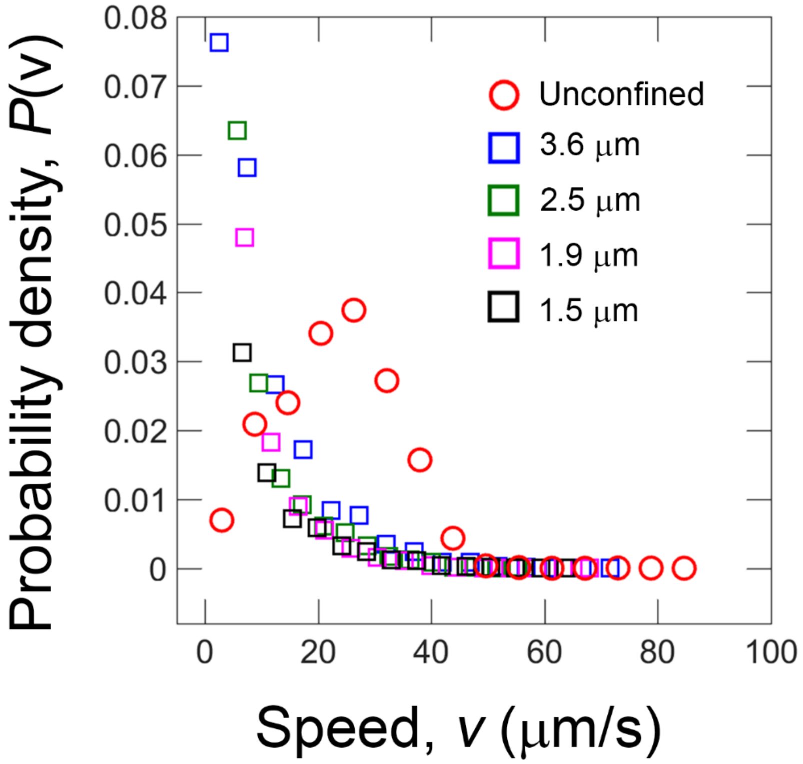

Supplementary Figure 6. Distribution of instantaneous speeds. We calculate the instantaneous speed of each cell as it moves through the pore space, $v(t) \equiv |\vec{\mathbf{r}}(t+\delta t) - \vec{\mathbf{r}}(t)|/\delta t$. We create discrete probability density functions for bacteria speed in unconfined liquid media as well as in different porous media. Probability density of all discrete speeds in unconfined media peaks at 28 μm/s. In porous media, the cell speeds are exponentially distributed with an increased probability density of smaller speeds due to pore-scale confinement.

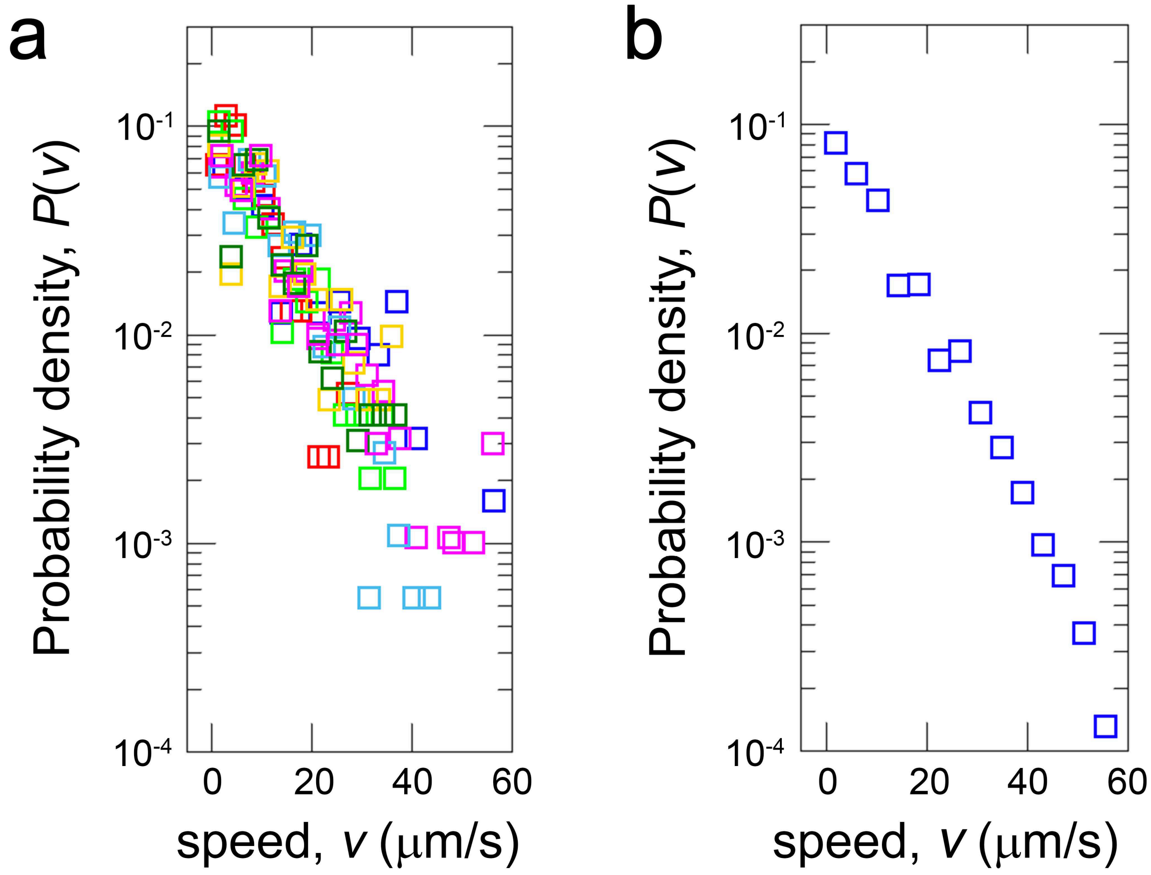

Supplementary Figure 7. Speed distributions of individual cells. To verify if the measured probability density function of instantaneous speeds reflects heterogeneous cellular responses, we plot the discrete PDF of speed for eight individual cells migrating through a porous medium with a characteristic pore size of 3.6 μm, shown by the different colors in panel (a). The PDFs are all similar, and are similar to the total PDF of the entire population (panel b), indicating that the variability in our measurements does not reflect the heterogeneous response of different cells, but rather, heterogeneity imparted by the disorder in the porous medium.

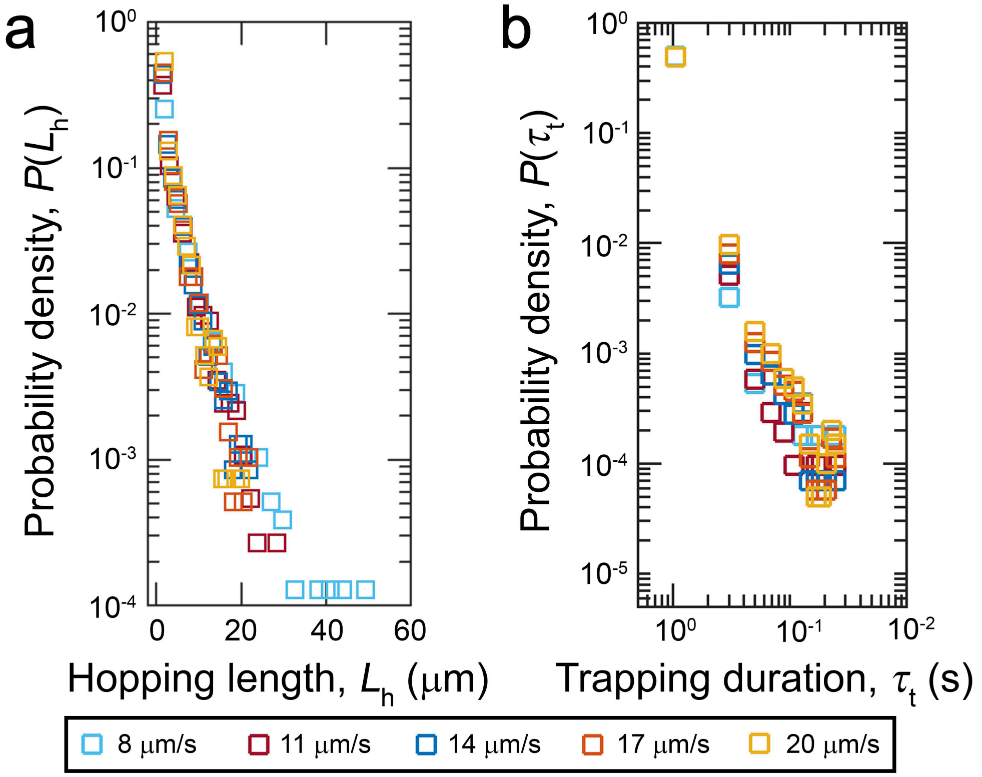

Supplementary Figure 8. Effect of speed cut-off. To verify if the distribution of trapped times and hopping times are affected by the choice the speed cutoff, we measure trapped times and hopping times using different speed cutoffs indicated by the legend for a porous medium with characteristic pore size 3.6 μm. We find that the choice of speed cutoff does not affect the distributions of hopping length and trap duration considerably, as shown by the different colors of points in panels a and b.

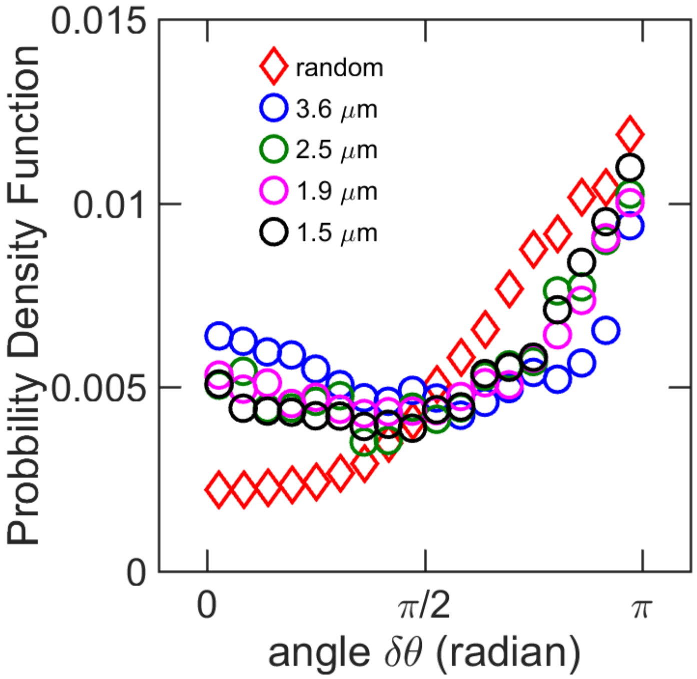

Supplementary Figure 9. Cells move in random orientations inside traps. Distribution of reorientation angle for all traps (circles); legend indicates characteristic pore sizes of the different media. We compare the measured distributions to a distribution of random points (diamonds): we generate an array of 10000 random points, measure the angle between the vectors connecting them, and plot the resultant distribution of angles. We find a similar trend, suggesting that trapped cells move in random orientations.

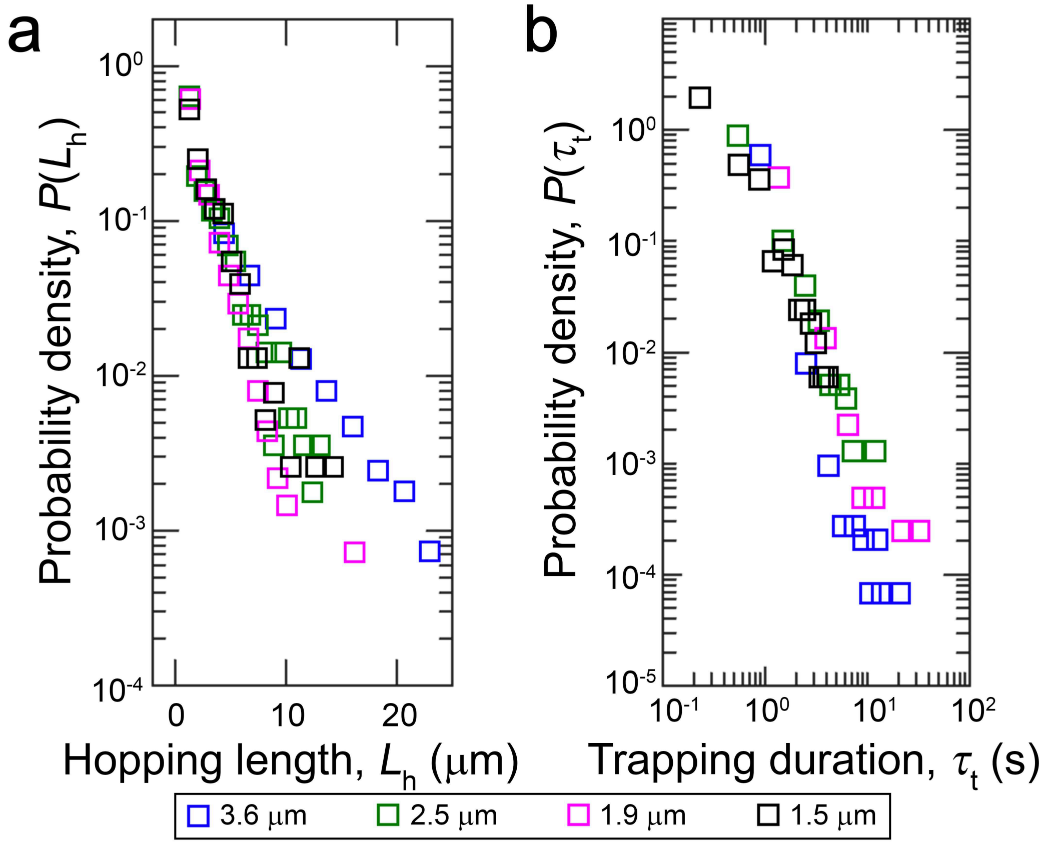

Supplementary Figure 10. Effect of minimum tracking duration. To test if the distribution of trapped times and hopping distances are affected by the choice of minimum tracking duration, we perform similar measurements including any motile cells that can be followed for a minimum of 1 s. We find similar hopping length and trapping duration distributions as those shown in Fig. 4 for cells with a minimum tracking duration of 10 s.

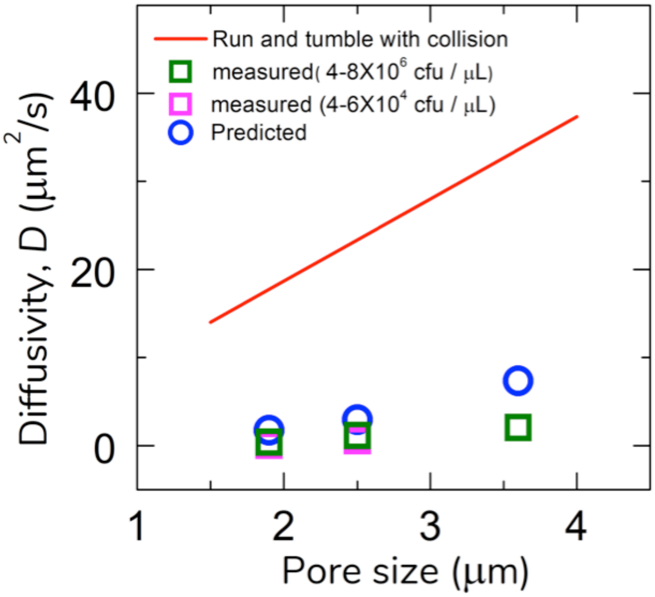

Supplementary Figure 11. Diffusivity measurements for different pore sizes and cell concentrations. To verify that the measurements of long-term diffusivity are not affected by the cell concentration in the bolus, we perform diffusivity measurements for two different cell concentrations within the starting bolus (green and magenta) and media with three different characteristic pore sizes. We find similar values of the measured diffusivity for both cell concentrations. We also note that our measured diffusivities are comparable to the prediction of the hopping-between-traps model (blue), while the run-and-tumble diffusivity with an unchanged run speed but a run length given by the characteristic pore size is over an order of magnitude off (red line).
