# Supplementary material for: Bacterial hopping and trapping in porous media
Source: Nat Commun. 2019 May 6;10:2075. doi: 10.1038/s41467-019-10115-1 (PMC6502825; doi:10.1038/s41467-019-10115-1)
Supplement: Supplementary file 3 — Description of Additional Supplementary Files [file 41467_2019_10115_MOESM3_ESM.pdf]

Description of Additional Supplementary Files

File Name: Supplementary Movie 1
Description: Intermittent hopping and trapping of *E. coli* in a 3D porous medium with a = 2.5 µm. Direct imaging reveals switching between two modes of motion: hopping, in which the cell moves through extended, directed paths through the pore space, and trapping, in which the cell is confined for extended periods of time.

File Name: Supplementary Movie 2
Description: *E. coli* with fluorescently-labeled flagella (magenta) in a 3D porous medium with a = 2.5 µm. Direct imaging shows that the cell becomes trapped when it encounters an obstruction; however, the flagella continue to rotate as a coherent bundle for much longer than the unconfined run duration. The cell continues to reorient itself while trapped, eventually enabling the flagella to unbundle and re-bundle in a different configuration, which enables the cell to escape its trap and continue to hop through the pore space in a different direction.

File Name: Supplementary Movie 3
Description: *E. coli* with fluorescently-labeled flagella (magenta) in a 3D porous medium with a = 2.5 µm. Direct imaging shows that the flagella remain bundled during cell trapping, and the cell leaves the trap only when the flagella can become transiently unbundled, further suggesting a correlation between trapping and flagellar bundling.
